# Flexible surge capacity – public health, public education, and disaster management

**DOI:** 10.34172/hpp.2020.30

**Published:** 2020-07-12

**Authors:** Amir Khorram-Manesh

**Affiliations:** Institute of Clinical Sciences, Department of Surgery, Sahlgrenska Academy, University of Gothenburg, Gothenburg, Sweden

**Keywords:** Disaster, Delivery of healthcare, Healthcare resources, Public health, Surge capacity

## Abstract

**Background:** Failed attempts to improve the delivery of healthcare to communities show distinct flaws that have a higher impact during a major incident or disaster (MID). This study evaluates the concept of surge capacity, which intends to achieve a balance between the needs and resources in affected areas by providing staff, stuff, structure, and system.

**Methods:** A systematic literature review was performed according to the PRISMA statement and by using PubMed, Scopus, and Google Scholar, and related keywords.

**Results:** There were limited publications about flexible surge capacity (FSC). However, the sum of data obtained indicated the need for flexibility in expanding major incidents or disasters, demanding new resources, which may neither be available on time nor reachable due to infrastructural damage.

**Conclusion:** FSC is a novel concept based on international guidelines. It refers to the extra and adjustable human and material resources that can be mobilized by activating nonprofessional but educated staff and different but accepted facilities in a fast, smooth, and productive way. Public health and public education play an essential role in obtaining such flexibility.

## Introduction

The current global socio-economic challenges, e.g., pandemic, terrorism, war, and armed conflicts, have resulted in the inability to support specific communities with necessary public services, and created “Underserved Communities” (UCs), particularly in disaster-affected countries (e.g., Thailand, the Philippines, etc).^1,2^ Failed attempts to improve the delivery of healthcare to communities show distinct flaws that have a higher impact during a major incident or disaster (MID), and result in an inability to anticipate, cope with, resist and recover from its consequences.^1,3-5^

A major incident is an event with a range of severe consequences, which can be managed by one or more emergency responder agencies and by reorganizing and rearranging of available resources. Disasters, on the other hand, are events that might not be controlled, causing considerable damage or loss of life. The change in the disaster management paradigm from reactive to proactive, necessitates greater respect for the social dimensions of disasters and an awareness of the underlying community resilience, appreciation, recognition, as well as strengthening of the local communities’ capacity, and the social and individual resiliency process they initiate and experience before and during MIDs.^1,3-6^ These steps require: a robust public emergency management plan; sufficient individual, social, and financial means; and necessary survival items.^6,7^

The concept of surge capacity (SC) in MID relates to the ability to increase staff, stuff, structure, and system (4S) rapidly and effectively in the affected areas. A secondary SC underlines the need for extra efforts to obtain additional resources.^8,9^ Nevertheless, a further expansion of the incident demands new approaches, policies, and adjustable preparedness within the community to scale up and down resources in a fast, smooth, and productive way, i.e., “flexible surge capacity” (FSC).

This narrative review aims to search for any evidence or plan for FSC in the literature, by which the community’s material and human resources can be activated or enhanced to mitigate or manage MIDs. Furthermore, it proposes and discusses the concept and implementation of FSC.

## Materials and Methods

**Figure 1:**
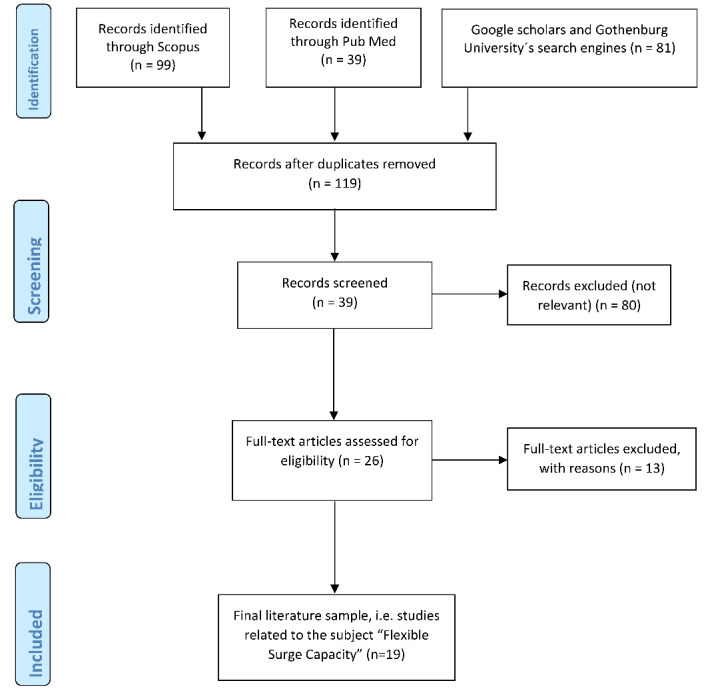
The process of literature search according to the PRISMA flow diagram.^10^

PubMed, Scopus, and Google Scholar search engines were used to identify publications related to the following keywords alone or in combination: Flexible, Surge Capacity, Disasters, Healthcare, Delivery, Emergencies, Community, Resources, and Public Health. The search started with one keyword, and additional keywords were added to reduce the number of hits. All initial abstracts were reviewed, and duplicates were removed. The inclusion criteria were publications dealing with FSC, i.e., measures implemented after primary and secondary surge capacity and within the community, published during the last 20 years. All non-relevant, non-scientific publications were excluded. Figure 1 shows the process of the literature search according to the PRISMA flow diagram.^10^

## Results

### Search results

Figure 1 presents the results of the search for each search engine.^10^ A total number of 219 publications were identified. Duplicates were removed (n = 100), and the author screened the remaining 119 publications. Eighty papers were not relevant to the subject, and in another 13 articles, FSC was mentioned, but no concept was presented or discussed. These papers were excluded. The remaining 26 documents were studied in detail, and the final sample (n = 19) was used in this study.

### Findings

*A. Why FSC? Risks and vulnerabilities as a foundation for FSC*

Flexible preparedness requires a correct risk and vulnerability analysis to identify strengths and weaknesses in our existing system. Such an analysis encompasses a proper methodology to identify hazards, which are dangers or risks. Risk is the overall summary of assets, threats, and vulnerabilities. Our assets are what we would like to protect in the community from threats that are manmade or natural incidents, e.g., terrorism, pandemic, or the outcome of climate change. Our vulnerabilities are the weaknesses that can be exploited by threats. These weaknesses can either be elements that are missing, such as strategic leadership, or those that exist in lower quality than needed, such as faulty risk and vulnerability analysis. The overall risk analysis would form the basis for further contingency planning, including the utilization of available or alternative resources, which would be the foundation of FSC.

The most critical factors leading to defective access to healthcare in the community are:

1. *New emerging risk factors:* New socio-economic and environmental risk factors increase the risk of new crises, which mainly affect the poorest and most marginalized groups of the community and necessitate new approaches to global health and emergency management.^7,11-13^
2. *Insufficient or limited essential public services:* The lack of basic public services affects people´s well-being and society´s functionality. Lack of specialized healthcare and guidelines and appropriate facilities create defects in healthcare delivery, particularly in UCs.^1,2,7^
3. *The failure of strategic leadership and sound decision-making at all managerial levels:* Strong and experienced guidance in all parts of the response chain is a necessity for successful emergency management.^14,15^Many aspects of leadership cannot be learned through leadership programs. Empowering families, women, and children as the smallest unit in society is the most critical mechanism to guarantee a sound and steady increase in welfare and to provide opportunities for the young generation, irrespective of gender, diversity, and origin to learn the needed skills for leadership and to enhance their hidden competencies.^16^
4. *Failed communication and collaboration with other stakeholders* : Failure to communicate and collaborate within one’s own organization and with others results in not only inadequate resource utilization but also the use of resources without control. As a result, the partnership with local non-governmental organizations (NGOs) and voluntary organizations would fail. Dental and veterinary professionals with a capacity to contribute either with human or material resources would not be used.^14,15,17-19^ Primary/community healthcare centers with competency in preventive services, ideally located in all parts of the community, and with the ability to improve patients´ resilience and ability to cope with unexpected emergencies would not be adequately utilized.^20-22^
5. *Hospitals’ shortcomings:* Hospitals’ emergency department overcrowding, poor strategic leadership, along with a shortage of devices and medical products, increased staff sub-specialization, and inadequate training in the management of MID, all result in defective healthcare.^14,17^
6. *Prehospital shortcoming:* Prehospital care and first responders, who are the essential link between victims and definitive medical care, suffer from either staff/resource shortages or delayed response time. The latter creates a gap in time, a therapeutic window which, if not used wisely, can result in poor management of a MID.^23^

*B. What to do? Proposing the concept of FSC*

There is a global need for increased and flexible resource utilization in a MID. Such flexibility demands collective and social responsibility and preparedness based on risk assessment, education, and the proper use of non-medical facilities.^24,25^ FSC refers to extra and adjustable human and material resources within a society, utilized by activating nonprofessional but educated staff, and different but accepted facilities. These untapped resources available within a community can be lifesaving and deserve recognition and support from all agencies within the emergency management network. Coordinating and leading these facilities and individuals is a viable strategy that needs policy change and a multidisciplinary dialogue, and is a more logical approach than creating new entities or professional staffs that may never face a MID.

*C. How to achieve FSC*

FSC emerges from societies consisting of people and governed by people working in different organizations such as public, private, and NGOs. These are the untapped resources within a community, which, if used wisely, can guarantee the accessibility and sustainability of healthcare and disaster management throughout all phases of a MID.

Public education is an essential part of FSC. According to the WHO recommendations, civilians should be empowered and promoted in self-dependency, first aid, and other simple procedures to quickly respond to a MID.^26,27^ Projects such as “Stop the bleed” in the United States, and “CitizenAID” in the United Kingdom, also aim to enable civilians to save lives by performing measures such as initial triage, and hemorrhage control.^28,29^ Other reports sought to define the concept and possible tasks for an “immediate responder” (bystander)^23^ and to establish an educational initiative to enable youth to act in an emergency.^30^ The idea is to let bystanders perform lifesaving measures, or at least learn how “Not to Harm.” Future studies aim to determine the content of, and the policies governing educational initiatives for the bystander. Nevertheless, they can also facilitate the concept of FSC.

According to a recent survey from the United States, none of the participating level I trauma center hospitals had enough critical care capacity or inpatient beds available to cope with a sudden influx from a mass casualty event.^21^ Many patients with a low priority could be examined and treated in nearby primary healthcare centers or could be observed in non-medical facilities by nurses and doctors with some necessary medical instruments and devices.^21,24,25^ Mapping common facilities and material resources may offer other options. *Schools and sports facilities* have basic healthcare routines; however, new threats such as school shootings indicate a need for an improvement in their medical capabilities to serve uninjured victims or vulnerable groups.^30^*Dental and veterinary clinics* have advanced medical knowledge and can easily take part in some defined activities during a MID. Their facilities and their equipment, such as CT-scans, can be used to address light injuries and for planned, non-urgent investigations.^11,18,19^*Primary Health centers* ,besides their proactive and informative role, may be able to handle either ambulatory surgical cases, or help hospitals’ surge capacity by accepting outpatients or non-disaster emergency cases. They may also handle specific cases either by receiving specialized staff and equipment from a hospital or by training a group of their own.^1,20-22^

*D. Who will manage FSC*

Public health is an interdisciplinary field that brings together sociology, psychology, other behavioral sciences, and clinical perspectives from medicine, nursing, dentistry, and nutrition. Public health professionals have skills in planning, administration, policy studies, biostatistics, and epidemiology. Together, they build up an extensive interdisciplinary team, which has the theoretical and methodological knowledge and skills needed to address a range of different tasks. These tasks may include preventing epidemics and spread of disease (e.g., COVID-19); protecting against environmental hazards and injuries; promoting and encouraging healthy behaviors and mental health; responding to disasters and assisting communities in recovery; and assuring the quality and accessibility of health services. Due to its presence throughout the MID, Public Health has a decisive and clear role in leading this process.^31^ It can coordinate, collaborate, and educate both the public and other professionals from diverse backgrounds (Emergency Medicine, Trauma, etc.), in developed or developing countries. It also has a significant role in changing social and cultural norms by increasing public awareness and conducting public education.^22,23,27,31^ The latter enables equal power relations, fosters individual skills while promoting community health, and sustainability, and prevents further divides in power relations. Analyzing the health of a population and the threats it faces, in peacetime as well as in a MID, is a critical task for public health, and facilitates a robust warning system to create relevant strategies and to save lives.^11,12,31^

## Discussion

Surge capacity (primary and secondary) is the first step required to respond to the imbalance between needs and resources during a MID.^11-14,17,23^ FSC is a novel concept, which offers an alternative to further scale up or down the MID management process by using civilians and other available entities and facilities.

Although the expansion of resources and facilities has been discussed in a few papers, no other study or review has proposed the concept of FSC before.^24,25^ This paper identifies a few, but important, causes of defective healthcare within an underserved community and addresses needed changes to accommodate the necessary readiness to mitigate or manage a disaster using community’s resources.

To achieve FSC, there is a need for robust public emergency management plan; sufficient individual, social, and financial means; new public policies, and educational initiatives within the community to scale up and down resources in a fast, smooth, and productive way. It also points out Public Health institutions as the most logical and competent institution to lead the whole process. However, new policies and other measures such as education, access, and empowerment need to be implemented to facilitate the concept of FSC. The concept of FSC is applicable and generalizable to all countries and scenarios, as it is evident from the results of previous studies as well as the current COVID-19 pandemic that proactive planning, and community resiliency are necessary for a successful management and outcome.^1,4,5-11,12,29-32^

Future studies are necessary to evaluate the concept of FSC by focusing on the following areas; 1) Regional and local capacity evaluation in collaboration with the public health authorities to identify available human and material resources within the community, 2) Identifying an alternative leadership at the hospital and prehospital levels in collaboration with regional disaster organizations, 3) Identifying a new model to exercise or simulate all phases of multiagency disaster management, based on real scenarios, to find out strengths and weaknesses of the organizations and to improve the access to all resources, and 4) To evaluate the ethical consequences of medical decision-making and triage outcome in all but particularly underserved communities.

### Limitations

This review is performed by searching publications in English and Swedish. Consequently, some important information published in other languages may have been missed. In addition, the search results have been evaluated by one author, which may cause a potential bias or imprecision. However, the reviewers’ assessment will minimize this risk. Finally, the concept, as presented in this paper, has not been presented or discussed in other papers and allows no comparing discussion of the obtained results.

## Conclusion

FSC is a novel concept based on international guidelines. It refers to the extra and adjustable human and material resources that can be mobilized by activating nonprofessional but educated staff and different but accepted facilities in a fast, smooth, and productive way. Public health and public education play an essential role in obtaining such flexibility. Public health encompasses areas as wide-ranging as disaster response, pandemic, and injury prevention. It is evident that by using knowledge, innovation, and education, and having the support needed from all involved organizations, public health would be able to lead and build a culture of safety and resilience at all levels, which is one of the priorities for action in disaster risk reduction and a necessity for FSC.^4,5^

## Ethical approval

Not applicable.

## Competing interests

None declared.

## Author’s contribution

The author designed, reviewed, wrote the draft, and submitted the paper.
